# Glycolipid-peptide conjugate vaccines enhance CD8^+^ T cell responses against human viral proteins

**DOI:** 10.1038/s41598-017-14690-5

**Published:** 2017-10-27

**Authors:** M. Speir, A. Authier-Hall, C. R. Brooks, K. J. Farrand, B. J. Compton, R. J. Anderson, A. Heiser, T. L. Osmond, C. W. Tang, J. A. Berzofsky, M. Terabe, G. F. Painter, I. F. Hermans, R. Weinkove

**Affiliations:** 1 Cancer Immunotherapy Programme, Malaghan Institute of Medical Research, Wellington, 6242 New Zealand; 2 Centre for Public Health Research, Massey University, Wellington, 6021 New Zealand; 3 The Ferrier Research Institute, Victoria University of Wellington, Lower Hutt, 5046 New Zealand; 4 Hopkirk Research Institute, Palmerston North, 4442 New Zealand; 5 Vaccine Branch, Center for Cancer Research, National Cancer Institute, National Institutes of Health, Bethesda, MD 20892 USA; 6 School of Biological Sciences, Victoria University of Wellington, Wellington, 6140 New Zealand; 7 Maurice Wilkins Centre, The University of Auckland, Auckland, 1142 New Zealand; 8 Wellington Blood & Cancer Centre, Capital & Coast District Health Board, Wellington, 6021 New Zealand; 9 Department of Pathology and Molecular Medicine, University of Otago Wellington, Wellington, 6021 New Zealand

## Abstract

An important goal of vaccination against viruses and virus-driven cancers is to elicit cytotoxic CD8^+^ T cells specific for virus-derived peptides. CD8^+^ T cell responses can be enhanced by engaging help from natural killer T (NKT) cells. We have produced synthetic vaccines that induce strong peptide-specific CD8^+^ T cell responses *in vivo* by incorporating an NKT cell-activating glycolipid. Here we examine the effect of a glycolipid-peptide conjugate vaccine incorporating an NKT cell-activating glycolipid linked to an MHC class I-restricted peptide from a viral antigen in human peripheral blood mononuclear cells. The vaccine induces CD1d-dependent activation of human NKT cells following enzymatic cleavage, activates human dendritic cells in an NKT-cell dependent manner, and generates a pool of activated antigen-specific CD8^+^ T cells with cytotoxic potential. Compared to unconjugated peptide, the vaccine upregulates expression of genes encoding interferon-γ, CD137 and granzyme B. A similar vaccine incorporating a peptide from the clinically-relevant human papilloma virus (HPV) 16 E7 oncoprotein induces cytotoxicity against peptide-expressing targets *in vivo*, and elicits a better antitumor response in a model of E7-expressing lung cancer than its unconjugated components. Glycolipid-peptide conjugate vaccines may prove useful for the prevention or treatment of viral infections and tumors that express viral antigens.

## Introduction

Eliciting proliferation and activation of cytotoxic CD8^+^ T cells that can destroy cells expressing pathogen-derived or tumor-associated peptides is an important goal in vaccine development^1,2^. However, unadjuvanted peptides fail to elicit strong cytotoxic T cell responses and, while live attenuated pathogens can generate CD8^+^ T cell-mediated immunity^3–5^, safety and manufacturing considerations favor synthetic adjuvants. Most adjuvants in clinical use, such as alum, oil-in-water emulsions, and Toll-like receptor (TLR) ligands^6–8^, have been approved based on their capacity to elicit antibody-mediated immune responses, not cytotoxic T cell responses. There is an urgent need for synthetic adjuvants capable of eliciting strong cytotoxic T cell responses.

While most existing adjuvants engage pattern recognition receptors (PRRs) within or on antigen-presenting cells (APCs), an alternative method of enhancing an immune response is to exploit the capacity of CD4^+^ T cells to provide ‘help’ to APCs^9^. For example, the keyhole limpet hemocyanin (KLH) carrier protein can activate CD4^+^ T cells to shape the cytotoxic T cell response against co-administered peptides^10,11^. However, such ‘non-cognate’ CD4^+^ T cell help generates weaker memory CD8^+^ T cell responses than does help from CD4^+^ T cells recognizing the same antigen^12,13^, while the latter approach may be constrained by difficulties identifying shared CD4^+^ and CD8^+^ epitopes given the extreme polymorphism of the major histocompatibility complex (MHC) in humans^14^.

Natural killer T (NKT) cells are a class of T cells expressing canonical T cell receptors that recognize glycolipid antigens presented by the non-classical MHC molecule, CD1d. NKT cells exist in a semi-activated state capable of responding rapidly to antigenic stimulation^15^. APCs express CD1d and are capable of processing and presenting glycolipids, such as the potent synthetic antigen α-galactosylceramide (α-GalCer), to NKT cells^16^. Similar to classical CD4^+^ T cell help, NKT cell help promotes dendritic cell (DC) licensing, maturation and the production of pro-inflammatory cytokines, *e.g*., IFN-γ and IL-12, which enhance CD8^+^ T cell responses against co-presented peptide antigens^17^. This help depends on peptide and glycolipid presentation by the same APC, and *in vivo* requires an interaction between CD40 and CD40L^18,19^. Potential advantages of exploiting NKT rather than conventional CD4^+^ T cell help in a clinical context include avoiding the need to select adjuvants according to MHC class II expression^20^, and eliciting a CD8^+^ T cell response with a distinct chemokine receptor profile^21,22^. In mouse models, NKT cell activation at the time of vaccination or infection promotes virus-specific CD8^+^ T cell memory^23,24^. Although there is abundant evidence of NKT cell adjuvant activity in murine models *in vivo*, evidence for an adjuvant effect in humans, who have much lower NKT cell frequencies, is much more limited.

We have previously synthesized glycolipid-peptide conjugate vaccines comprising an α-GalCer prodrug covalently linked to an antigenic peptide via a self-immolating para-aminobenzyl (PAB) linker. These vaccines were designed to co-deliver CD8^+^ T cell peptide epitopes and α-GalCer to the same APC and elicit functional peptide-specific CD8^+^ T cell responses in mouse models of allergic airway inflammation and B.16 melanoma^25,26^. We have also shown that a glycolipid-peptide conjugate vaccine incorporating α-GalCer with an immunodominant HLA-A*02-restricted peptide from the cytomegalovirus (CMV) pp65 protein can stimulate human NKT cells and peptide-specific CD8^+^ T cells^25^.

Here we investigate the activity and mechanism of a glycolipid-peptide conjugate vaccine that elicits virus-specific CD8^+^ T cell responses in human PBMCs. We show that this vaccine activates human DCs and CD8^+^ T cells in a manner dependent on NKT cells, and that the CD8^+^ T cells elicited by this vaccine are peptide-specific and have cytotoxic potential. Furthermore, we show that a vaccine of the same design but incorporating a human papilloma virus (HPV) E7 peptide is capable of delaying tumor growth in an *in vivo* mouse model of E6/E7-expressing lung cancer.

## Results

### Glycolipid-peptide conjugate vaccine requires cathepsin cleavage and induces CD1d-dependent NKT cell proliferation

**Figure 1:**
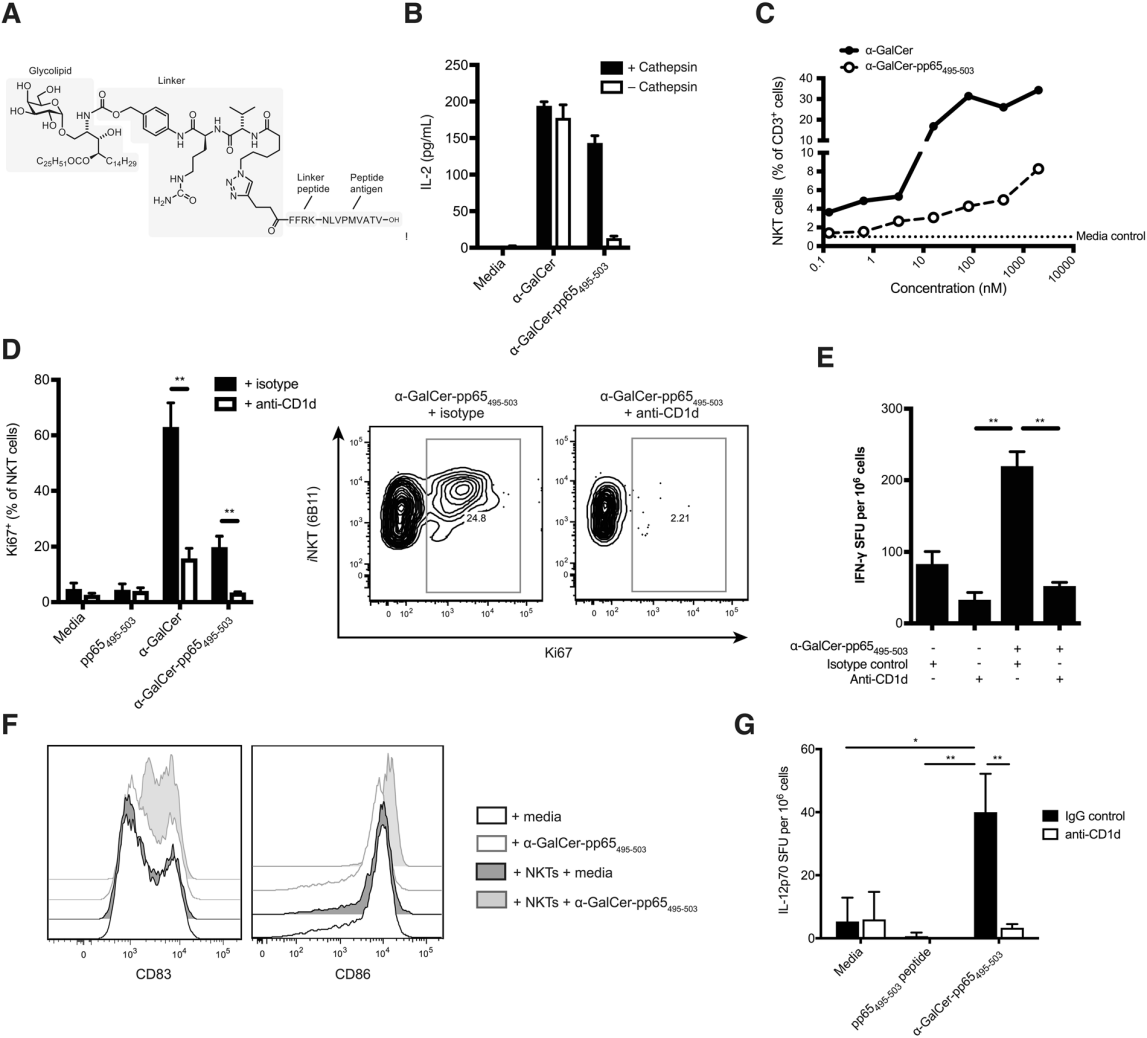
α-GalCer-pp65_495-503_ conjugate vaccine activates human NKT cells and DCs (**A**) Chemical structure of the conjugate vaccine, α-GalCer-pp65_495-503_, containing the HLA-A*02-restricted ‘NLV’ peptide from cytomegalovirus pp65 protein linked via an enzymatically cleavable linker to a pro-α-GalCer (**B**) IL-2 production by mouse NKT hybridoma cells was measured by enzyme-linked immunosorbent assay (ELISA) 18 h after addition of equimolar concentrations of α-GalCer or α-GalCer-pp65_495-503_ pre-treated with cathepsin-B or PBS **p < 0.01; Bonferroni multiple comparison test. (**C**) The number of NKT cells (% of total CD3^+^ cells) was quantified by flow cytometry in PBMCs from a HLA-A*02 negative donor 72 h after addition of varying concentrations of α-GalCer or α-GalCer-pp65_495-503_; representative of two independent experiments. (**D**) Proliferation of NKT cells was measured by flow cytometry using anti-Ki67 72 h after treatment of PBMCs from a HLA-A*02 negative donor with equimolar concentrations of pp65_495-503_ peptide, α-GalCer, or α-GalCer-pp65_495-503_ with anti-CD1d or matched isotype control antibody **p < 0.01; Bonferroni multiple comparison test. Data representative of two independent experiments. (**E**) IFN-γ production was measured by ELISpot 72 h after treatment of PBMCs from a HLA-A*02 negative donor with α-GalCer-pp65_495-503_+/− anti-CD1d or matched isotype control antibody **p < 0.01; Student’s T test; SFU, spot-forming units. (**F**) Expression of the activation markers CD83 and CD86 on monocyte-derived (mo)DCs derived from a HLA-A*02 negative donor 48 h after treatment with α-GalCer-pp65_495-503_ or media control, in the presence or absence of autologous NKT cells. Result representative of three independent experiments.

The glycolipid-peptide conjugate vaccine α-GalCer-pp65_495-503_ (Fig. 1A) consists of a pro-drug form of the glycolipid α-galactosylceramide (α-GalCer), which readily reverts to its more stable N-acyl form under physiological conditions^25^, linked via a cathepsin-B-cleavable linker to the peptide sequence FFRK-NLVPMVATV (here termed pp65_495-503_), which contains a HLA-A*02-restricted epitope from cytomegalovirus (CMV) pp65 protein. CD8^+^ T-cells specific for NLVPMVATV can be readily detected in PBMCs from HLA-A*02^+^ CMV-seropositive healthy donors using loaded MHC class I multimers^27^. The peptide sequence incorporates the cleavage sequence FFRK at the N-terminus to promote proteolytic generation of the NLVPMVATV epitope within APCs^28^.

To show that conjugate vaccine must first be cleaved into its active components in order to stimulate NKT cells, α-GalCer-pp65_495-503_ and free α-GalCer were pre-treated with cathepsin-B or PBS control, and loaded onto plate-bound mouse CD1d monomers. Unlike free α-GalCer, α-GalCer-pp65_495-503_ required pre-treatment with cathepsin-B in order to stimulate IL-2 production by the mouse hybridoma NKT cell line DN32.3, indicating that the α-GalCer-pp65_495-503_ vaccine requires proteolytic processing to produce free α-GalCer capable of activating NKT cells (Fig. 1B).

We have previously shown that α-GalCer-pp65_495-503_ is able to induce IFN-γ production and CD137 up-regulation on human NKT cells^25^. To determine whether α-GalCer-pp65_495-503_ can also induce proliferation of NKT cells, PBMCs derived from an HLA-A*02-negative donor were cultured in the presence of equimolar concentrations of α-GalCer or α-GalCer-pp65_495-503_ conjugate. Quantification of NKT cells (as a % of total CD3^+^ cells) showed that addition of α-GalCer-pp65_495-503_ induced NKT cell expansion in a dose-dependent manner, although overall expansion was lower with the vaccine than with free α-GalCer (Fig. 1C). Similarly, intracellular staining using anti-Ki67 showed proliferation of NKT cells in response to both α-GalCer-pp65_495-503_ and to free α-GalCer, which could be abolished by addition of an anti-CD1d antibody (Fig. 1D). As expected, the peptide alone did not trigger NKT cell proliferation above the level of the media-only control. Finally, interferon (IFN)-γ ELISpot demonstrated that α-GalCer-pp65_495-503_ induced IFN-γ production, and that this was blocked by anti-CD1d (Fig. 1E). Taken together, these data demonstrate that the glycolipid-peptide conjugate vaccine α-GalCer-pp65_495-503_ induces proliferation and activation of human NKT cells in a CD1d-dependent manner.

In mice, the presentation of α-GalCer by DCs on CD1d activates NKT cells to ‘license’ DCs, in a manner analogous to traditional CD4^+^ T cell help^17^. This leads to up-regulation of DC co-stimulatory markers and increased IL-12 production, which further activates NKT cells, as well as augmenting peptide-specific CD8^+^ T cell responses^29–31^. To assess the ability of α-GalCer-pp65_495-503_ to activate human DCs, expression of the maturation marker CD83 and co-stimulatory molecule CD86 was assessed by flow cytometry 48 h after addition of α-GalCer-pp65_495-503_ to a co-culture of human moDCs and autologous NKT cells expanded *ex vivo* (Fig. 1F). α-GalCer-pp65_495-503_ did not induce DC activation in the absence of NKT cells indicating that it does not directly engage PRRs. Instead, only moDCs treated with α-GalCer-pp65_495-503_ and co-cultured with NKT cells showed elevated expression of CD83 and CD86 above levels seen on unsupplemented moDCs. Similar results were seen using unconjugated α-GalCer (data not shown). Upon presentation of α-GalCer to NKT cells, murine dendritic cells produce IL-12^29^. To determine whether the α-GalCer-pp65_495-503_ conjugate can induce IL-12 production by human APCs, PBMCs from an HLA-A*02-negative donor were cultured with pp65 peptide, α-GalCer, α-GalCer-pp65_495-503_ conjugate or media control in the presence of anti-CD1d antibody or IgG control; IL-12p70 production was determined by ELISpot (Fig. 1G **)**. Both α-GalCer and α-GalCer-pp65_495-503_ led to CD1d-dependent IL-12p70 production, consistent with DC activation. Thus, α-GalCer-pp65_495-503_ activates human DCs in an NKT cell- and CD1d-dependent manner.

### Glycolipid-peptide conjugate vaccine leads to peptide-specific CD8^+^ T cell activation

**Figure 2:**
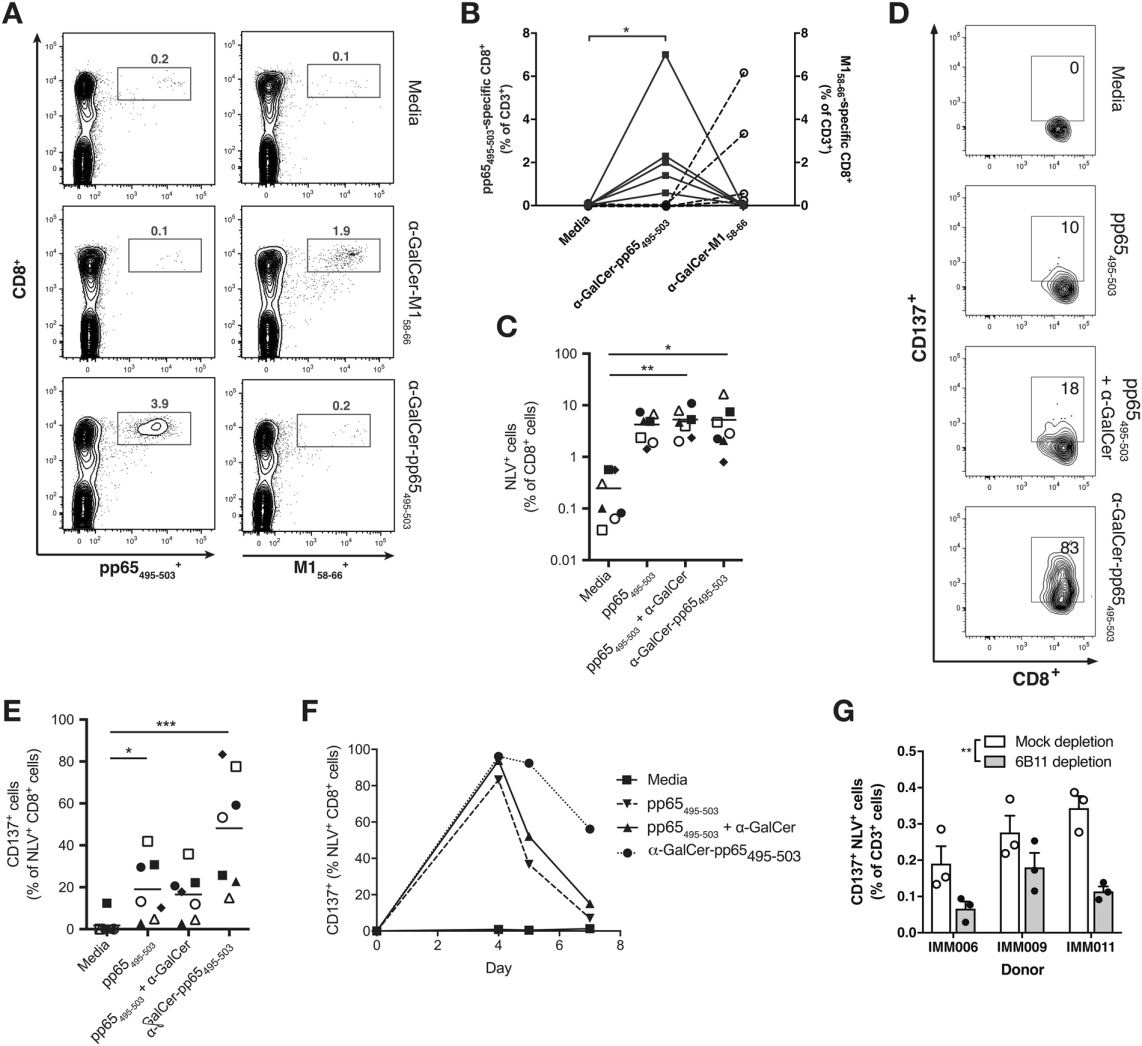
α-GalCer-pp65_495-503_ activates NLV-specific CD8^+^ T cells in an NKT cell-dependent manner (**A**, **B**) Peptide specificity of the CD8^+^ T cell pool expanded with α-GalCer-pp65_495-503_, or with a similar conjugate vaccine containing an influenza matrix protein-derived peptide (α-GalCer-M1_58-66_) was demonstrated measured by dual dextramer staining; representative flow cytometry plots (**B**) and summary of results of five HLA-A*02^+^ CMV-responsive donors tested (**B**) are shown. Square symbols and solid lines represent pp65_495-503_-loaded tetramer^+^ cells; black open circles and dashed lines represent M1_58-66_-loaded tetramer^+^ cells; *p < 0.01; Friedman test with Dunn’s multiple comparison tests. Frequency (**C**) and CD137 expression (**D**,**E**) of NLV-specific CD8^+^ T cells was quantified by flow cytometry 7 days after adding equimolar concentrations of pp65_495-503_ peptide alone, peptide admixed with α-GalCer or α-GalCer-pp65_495-503_ to PBMCs from HLA-A*02^+^ donors. *p < 0.05, **p < 0.01, ***p < 0.001; Friedman test with Dunn’s multiple comparison tests. Representative flow cytometry plots are shown in (**D**). (**F**) Time-course showing CD137 status of NLV-specific CD8+ T cells 4–7 days after exposure to pp65_495-503_ peptide, peptide admixed with α-GalCer or α-GalCer-pp65_495-503_; result representative of two independent experiments. (**G**) PBMCs were depleted of NKT cells by immunomagnetic beads, or mock depleted, and CD137^+^ status of NLV-specific CD8^+^ T cells was quantified by flow cytometry 7 days after addition of α-GalCer-pp65_495-503_, in triplicate. The results from three separate donors in three separate experiments are shown. ***p < 0.001 for an effect of NKT cell depletion by two-way ANOVA; **p < 0.01, Bonferroni’s post tests.

We have previously shown that the α-GalCer-pp65_495-503_ conjugate vaccine can induce activated peptide-responsive CD8^+^ T cells^25^. To verify the peptide specificity of CD8^+^ T cell activation, we made an alternative conjugate vaccine incorporating the immunodominant HLA-A*02-restricted peptide from influenza A virus (IAV) matrix-1 (M1) protein, here termed α-GalCer-M1_58-66_. Dual dextramer staining of PBMCs treated with α-GalCer-pp65_495-503_ or α-GalCer-M1_58-66_ showed that the α-GalCer-pp65_495-503_ conjugate vaccine induced expansion of pp65_495-503_-specific but not of M1_58-66_-specific CD8^+^ T cells, and *vice versa* for α-GalCer-M1_58-66_ (Fig. 2A,B), confirming that the activation of CD8^+^ T cells by glycolipid-peptide conjugate vaccines is peptide-specific.

We assessed the capacity of the α-GalCer-pp65_495-503_ conjugate vaccine to stimulate peptide-specific CD8^+^ T cell responses using PBMCs from HLA-A2^+^ CMV seropositive donors known to harbor a pre-existing population of NLVPMVATV- (NLV; pp65_495-503_) specific CD8^+^ T cells in three donors previously reported^25^ and four additional donors. Among these donors, a median of 0.08% of CD3^+^ T cells were NKT cells (range 0.02–0.35%), as assessed by expression of the Vα24 Jα18 TCR. After seven days of culture, the proportion of peptide-specific CD8^+^ T cells (as a % of total CD8^+^ cells) detectable with fluorescent NLV-loaded HLA-A2 dextramers was significantly increased in cultures containing α-GalCer-pp65_495-503_ compared to unsupplemented (media-only) controls (Fig. 2C). In this *in vitro* system, the total number of NLV-specific CD8^+^ T cells did not differ between cultures treated with α-GalCer-pp65_495-503_, its admixed components or the pp65_495-503_ peptide alone.

After seven days in culture NLV-specific CD8^+^ T cells expanded with α-GalCer-pp65_495-503_ showed higher expression of the T cell activation marker CD137 (4-1BB), a surface glycoprotein belonging to the tumor-necrosis factor receptor superfamily (TNFRSF)^32,33^, compared to cells treated with either peptide alone or admixed peptide and α-GalCer (Fig. 2D,E). Binding of CD137 to its ligand 4-1BBL promotes increased T cell proliferation, cytokine production, functional maturation, and prolonged survival of CD8^+^ T cells^34–36^. Interestingly, at earlier time-points CD137 expression was also elevated in response to admixed peptide and α-GalCer as well as peptide alone, however, this elevated expression was only sustained on NLV-specific CD8^+^ T cells expanded with α-GalCer-pp65_495-503_, suggesting that conjugation modifies the T cell response to peptide or affects the kinetics of peptide presentation (Fig. 2F).

To determine whether NKT cells are required for the CD8^+^ T cell response to α-GalCer-pp65_495-503_, NKT cells were depleted from whole PBMCs derived from three separate donors. Compared to mock-depleted controls, NKT depletion led to a significant reduction in the proportion of CD137^+^ NLV-specific T cells in response to α-GalCer-pp65_495-503_ (Fig. 2G), indicating that full vaccine-induced activation of peptide-specific CD8^+^ T cells requires NKT cells.

### Glycolipid-peptide conjugate elicits peptide-specific CD8^+^ T cells with cytotoxic potential

**Figure 3:**
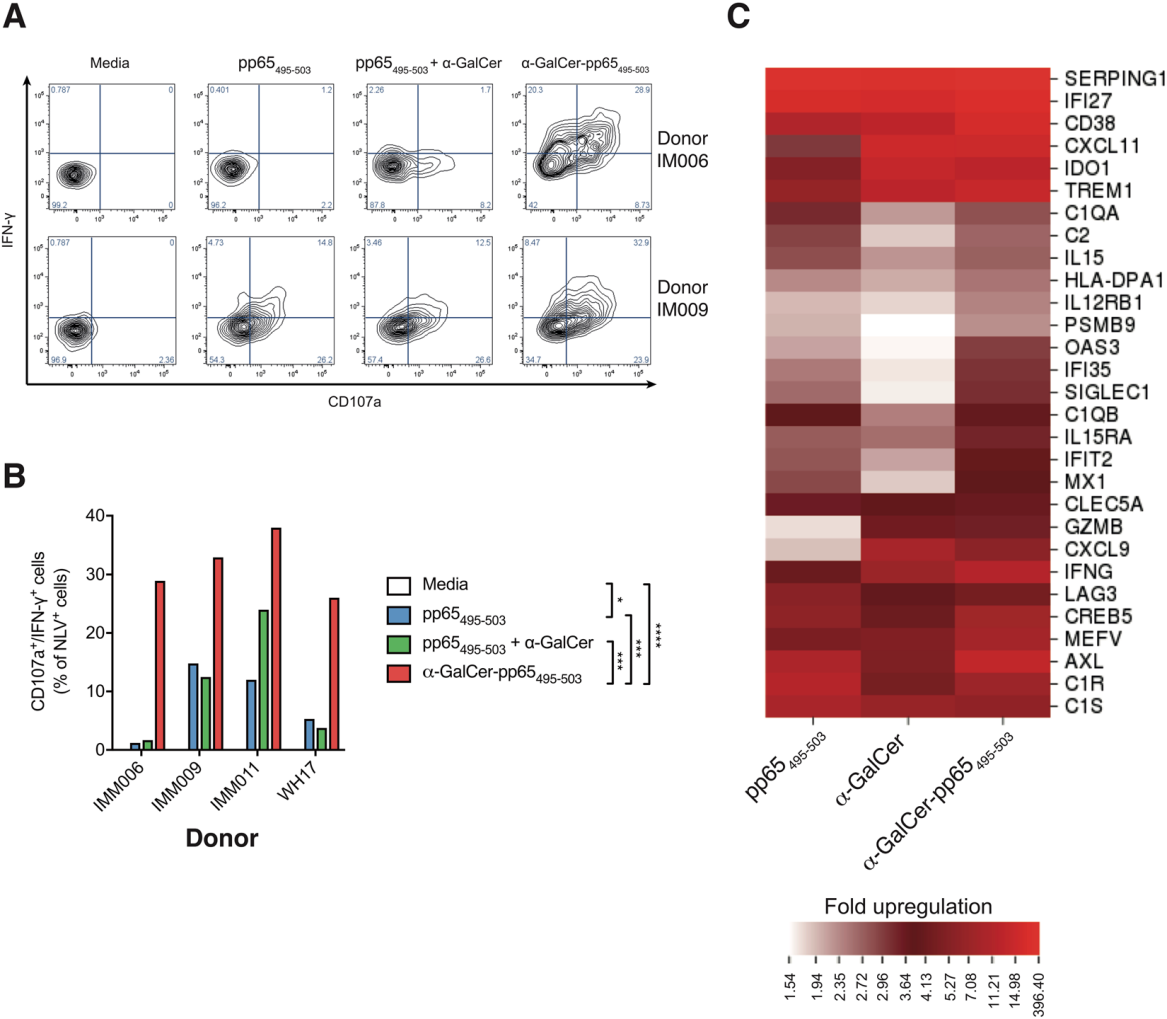
α-GalCer-pp65_495-503_ elicits a cytotoxic CD8^+^ T cell phenotype and expression of interferon-inducible genes. (**A**) Representative flow plots and (**B**) summary graph from four donors showing expression of CD107a and IFN-γ by NLV-specific CD8^+^ T cells 7 days after addition of equimolar concentrations of pp65_495-503_ peptide alone, pp65_495-503_ admixed with α-GalCer or the α-GalCer-pp65_495-503_ conjugate. ****p < 0.0001; ***p < 0.001; *p < 0.05, two way ANOVA with Tukey’s multiple comparisons test. (**C**) Nanostring RNA analysis was performed on whole PBMCs from human donors (n = 4) treated with α-GalCer-pp65_495-503_, pp65_495-503_ peptide, or α-GalCer for 7 days. Heatmap shows the genes at least two-fold up-regulated in response to α-GalCer-pp65_495-503_, compared with media alone, in all four donors.

To assess the functional outcome of α-GalCer-pp65_495-503_ treatment, intracellular staining for LAMP-1 (CD107a), a marker of degranulation of cytotoxic molecules, and the pro-inflammatory cytokine IFN-γ was performed on NLV-specific CD8^+^ T cells. Both LAMP-1 and IFN-γ were expressed to a higher degree on NLV-specific CD8^+^ T cells expanded with α-GalCer-pp65_495-503_, compared to those exposed to peptide alone, or to admixed peptide and α-GalCer (Fig. 3A,B). In contrast, α-GalCer-pp65_495-503_ did not induce expression of LAMP-1 or IFN-γ on the dextramer negative CD8^+^ T cell population within the cultures (data not shown), indicating that the induction of these molecules is restricted to antigen-specific T cells.

To further explore the effect of α-GalCer-pp65_495-503_ treatment on human PBMCs, Nanostring RNA analysis was performed on RNA derived from whole PBMCs after seven days of culture with α-GalCer-pp65_495-503_ conjugate, its components (pp65_495-503_ peptide or α-GalCer), and media control (Fig. 3C). α-GalCer-pp65_495-503_ led to elevated expression of the interferon-inducible genes *IFI35*, *OAS3*, *IFIT2*, and *MX1*, compared with levels seen in response to either peptide alone, α-GalCer alone or media control. Compared to pp65_495-503_ peptide, the α-GalCer-pp65_495-503_ conjugate led to higher expression of the genes encoding interferon gamma-induced protein-10 (IP-10; *CXCL10*), IFN-γ (*IFNG*) and CD137 (*TNFRSF9*), consistent with the phenotypic findings of increased IFN-γ and CD137 expression (Supplementary Table 1). Although upregulation of *LAMP1* was not observed at the RNA level (mean 1.1 fold expression in α-GalCer-pp65_495-503_- compared to pp65_495-503_-treated cells), expression of the cytotoxin granzyme B (*GZMB*) was enhanced by the conjugate vaccine.

Taken together, these data indicate that the α-GalCer-pp65_495-503_ glycolipid-peptide conjugate vaccine can elicit peptide-specific T cells with cytotoxic potential in human PBMCs.

### Glycolipid-peptide conjugate elicits cytotoxic responses to a viral antigen *in vivo*

**Figure 4:**
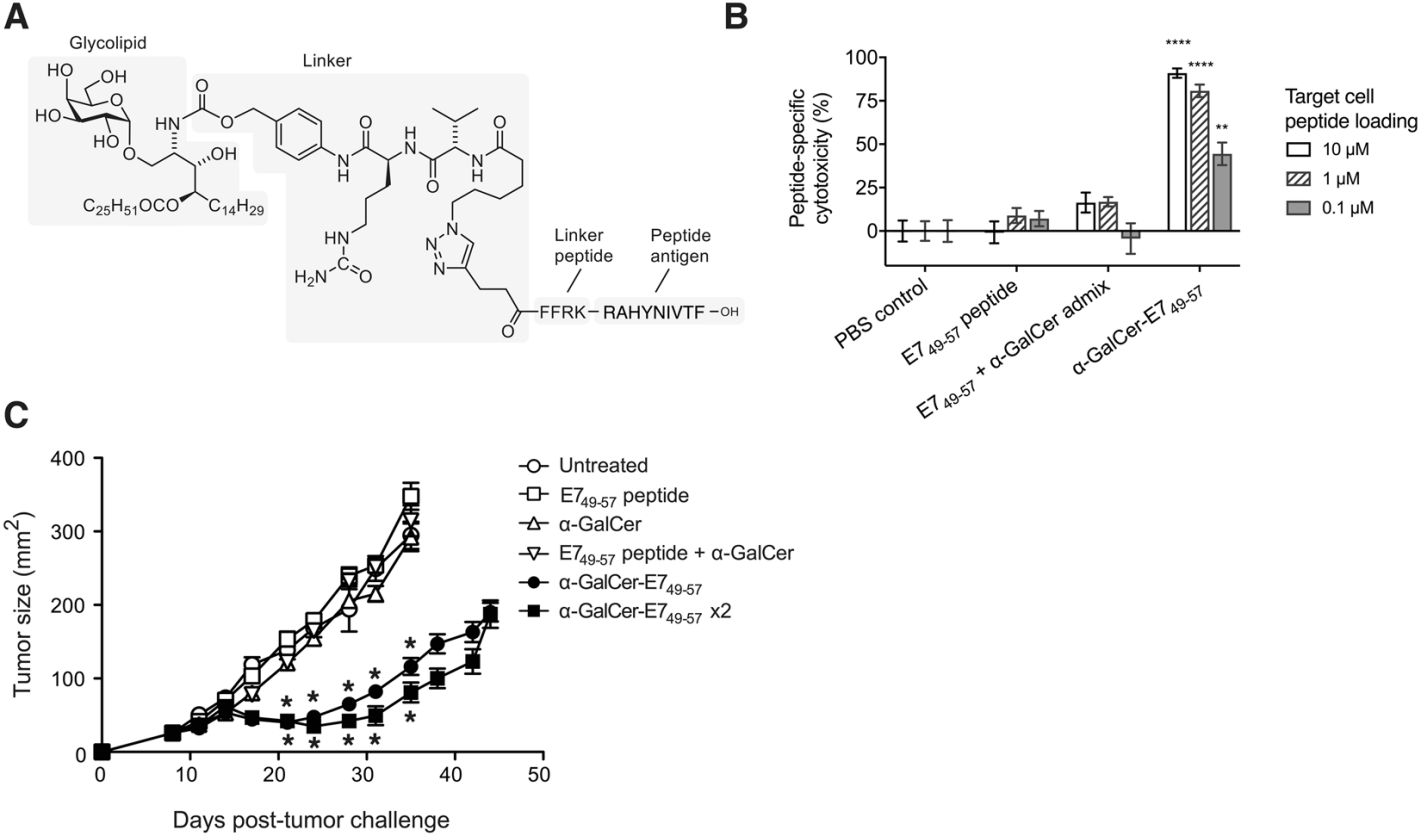
α-GalCer-E7_49-57_ conjugate vaccine delays growth of an established HPV16-E7-expressing tumor *in vivo*. (**A**) Chemical structure of the conjugate vaccine, α-GalCer-E7_49-57_, incorporating the RAHYNIVTF peptide from HPV16-E7 protein (**B**) C57BL/6 mice (5 per group) were vaccinated intravenously with α-GalCer-E7_49-57_, α-GalCer and peptide admix, E7_49-57_ peptide alone or PBS control. Specific lysis of E7_49-57_ peptide-loaded splenocytes was assessed *in vivo* 10 days after vaccination. ****p < 0.0001; **p < 0.01, one-way ANOVA with Tukey’s multiple comparisons (**C**) 2.5 × 10^5^ HPV16-E6 and -E7 protein-expressing TC-1 cells were implanted subcutaneously into C57/BL6 mice (n = 5 per group). On day 8, E7_49-57_ peptide, α-GalCer, admix or α-GalCer-E7_49-57_ were administered intravenously; one group was left untreated, and one group received a second dose of α-GalCer-E7_49-57_ after a further seven days. Tumor size was determined as the product of the two diameters. *p < 0.0001 (difference between treatment group, and each of the four non-conjugate vaccine groups) by one-way ANOVA.

To assess whether conjugate vaccines elicit peptide-specific cytotoxicity against viral antigens *in vivo*, conjugate vaccine α-GalCer-E7_49-57_ (Fig. 4A **)** was synthesized using the H-2D^b^-restricted peptide RAHYNIVTF from HPV16-E7 protein, an oncoprotein associated with HPV-driven malignancies including cervical and head and neck cancers. Again, FFRK was added to the N-terminus of the viral sequence to promote proteolytic release of the minimal MHC-binding peptide.

To assess capacity of this vaccine to induce peptide-specific responses, mice were vaccinated with α-GalCer-E7_49-57_, admixed α-GalCer and E7_49-57_ peptide or saline control. Splenocytes were restimulated *ex vivo* with the E7_49-57_ peptide, and IFN-γ production assessed by ELISpot. Compared to its admixed components or saline control, α-GalCer-E7_49-57_ led to increased peptide-induced IFN-γ production (Supplementary Fig. S1). To determine whether the vaccine could induce cytotoxicity against peptide-expressing targets *in vivo*, animals were vaccinated with α-GalCer-E7_49-57_, α-GalCer and peptide admix, E7_49-57_ peptide alone, or saline control, then E7_49-57_ peptide-pulsed or unpulsed target splenocytes were administered, and their frequency assessed by flow cytometry. The results indicate that the α-GalCer-E7_49-57_ vaccine induces *in vivo* cytotoxicity against cells expressing a virus-derived peptide (Fig. 4B).

To assess functional activity, α-GalCer-E7_49-57_ vaccine was administered to animals implanted with cells from the TC-1 tumor cell line, which expresses HPV16-E6 and -E7 proteins. A single dose of α-GalCer-E7_49-57_ vaccine eight days after tumor inoculation significantly delayed tumor growth relative to saline- or unconjugated component-treated control animals. This activity was improved further with a second dose of vaccine 7 days after the first. The antitumor activity was dependent on the vaccine design, with antigen and adjuvant covalently linked together, as injection of unconjugated components alone or as an admix did not provide antitumor activity **(**Fig. 4C **)**.

## Discussion

We have previously shown that synthetic vaccines that combine an NKT-activating glycolipid with an immunodominant antigenic peptide are effective against a model antigen (ovalbumin) *in vivo*, and that a similar vaccine can activate human CMV-specific CD8^+^ T cells *in vitro* ^25,26^. He we report that the activation of human virus-specific CD8^+^ T cells induced by these vaccines is NKT cell-dependent and peptide-specific, and that the vaccines activate human antigen-presenting cells in the presence of NKT cells, elicit peptide-specific CD8^+^ T cells with cytotoxic potential, and induce cytotoxicity against a clinically-relevant viral oncoprotein *in vivo*.

To-date, most pre-clinical studies using NKT cell agonists to drive peptide-specific T cell or antibody responses have been performed in mice, which have much higher circulating NKT cell frequencies than most humans^37^. Although human NKT cell frequencies typically exceed those of a CD4^+^ T cell of a given peptide specificity^38,39^, the reduced NKT cell niche has been raised as a barrier to generating effective immunotherapies utilizing these cells^40^. The current study provides “proof of principle” that human NKT cells can be successfully recruited to augment cytotoxic CD8^+^ T cell responses, despite their low frequency.

An important component of the α-GalCer-pp65_495-503_ vaccine is the cathepsin-B-sensitive linker, the removal of which permits an O→N acyl migration to form α-GalCer. As the glycolipid component of the vaccine must reach the late endosomal or lysosomal compartment for loading onto CD1d^41^, the requirement for this extra processing step may explain why α-GalCer-pp65_495-503_ was a less effective NKT cell agonist than free α-GalCer *in vitro*. However, given that repeated systemic α-GalCer injections have been shown to drive long-term NKT cell functional anergy arising from the development of an IL-10-secreting NKT cell subset (NKT_10_)^42,43^, this reduced activity may be advantageous *in vivo* at preventing NKT cell over-activation from stymying the downstream CD8^+^ T cell response or inducing tissue damage in the liver and other tissues where NKT cells are prevalent.

Crucially, this study uses ‘real-life’ viral antigens, rather than a model antigen, such as ovalbumin. The CMV epitope was selected based on the ability to detect CD8^+^ T cells specific for this virus-derived peptide at a high frequency in a large proportion of human donors^27^. Due to the persistent nature of CMV infection, CMV-reactive T cells have an altered phenotype compared with other virally-reactive or naïve tumor-associated antigen (TAA)-reactive T cells^44^. For example, CMV-specific CD8^+^ T cells are chronically activated and express the T_H_1-associated transcription factors T-bet (*TBX21*) and eomesodermin (*EOMES*), as well as *IFNG* mRNA and IFN-γ–regulated genes^45^. We cannot therefore be certain that the responses generated against the immunodominant CMV antigen will extend to other viral- or tumor-associated peptides, although it is reassuring that we observe CD8^+^ T cell responses to an immunodominant influenza matrix protein epitope using a similar vaccine.

The human cell *in vitro* culture system we used showed no difference in the frequency of NLV-specific CD8^+^ T cells between cultures treated with α-GalCer-pp65_495-503_, its admixed components or peptide alone, and while we demonstrate upregulation of molecules associated with cytotoxic function in response to the conjugate vaccine, *in vitro* killing of peptide-loaded targets is not demonstrated here. *In vitro* immunological studies are limited by the lack of a tertiary lymphoid structure that facilitates juxtaposition of the various immune cells involved^46^. Additional challenges to working with primary human cells *in vitro*, include: (1) the restricted number of professional, cross-presenting APCs and NKT cells present in each well; (2) the enforced proximity inherent in cell culture conditions, which all but ensures co-delivery of antigen and adjuvant to the same APC, regardless of conjugation; and (3) the substantial degree of heterogeneity between humans, which makes it difficult to detect differences within a small donor pool. Differences between peptide and conjugated vaccines may also be understated in *in vitro* studies due to the role of biodistribution in determining the nature and magnitude of the downstream immune response. For example, α-GalCer is known to bind apolipoprotein E (ApoE), enabling it to be efficiently acquired by DCs, via low-density lipoprotein (LDL) receptors^47^. Notably our data do show that a similar conjugate vaccine can induce cytotoxicity and delay tumour growth *in vivo*.

Oncogenic viral antigens are likely to be excellent targets for immunotherapeutic vaccine strategies aimed at reinforcing tumor-specific T-cell responses^48^. A recent clinical study in patients with HPV16^+^ high-grade vulvar intraepithelial neoplasia showed that vaccination with peptides in montanide, an oil emulsion that improves antigen uptake, could induce partial or complete histological regression of lesions in more than 50% of patients treated^49^. Importantly, this trial reported that clinical efficacy was related to the strength of vaccine-induced immune responses. It is conceivable that the outcome could be improved further with a vaccine that is designed to improve the function of APCs, as has been described here. The antitumor response reported here in mice against a tumor expressing the HPV16 E7 protein is notable both for the low dose of vaccine need for activity, and the fact that activity was reliant on conjugation of the glycolipid and peptide components. Studies of this vaccine design with other tumor-associated antigens, and in models of viral infection, are ongoing.

In conclusion, we show that in human PBMCs a glycolipid-peptide conjugate vaccine can harness NKT cells as cellular adjuvants to enhance cytotoxic CD8^+^ T cell responses against virus-derived peptides. We have demonstrated that a similar conjugate vaccine directed against an oncogenic viral protein is capable of generating a significantly better anti-tumor response than its admixed components in a model of HPV-associated cancer *in vivo*. Human trials will be necessary to determine whether glycolipid-peptide conjugates can safely enhance peptide-specific CD8^+^ T cell responses with clinical benefit.

## Methods

### Conjugate vaccine synthesis

Conjugate vaccines were synthesized per previously reported protocols^25^. Briefly, α-GalCer was treated with hydrochloric acid in dioxane and heated to 65 °C to reveal the phytosphingosine amine which was subsequently capped with the linker group N-(6-azidohexanoyl)-valine-citruline-4-aminobenzyl 4-nitrophenyl carbonate. The azido group was subsequently conjugated to N-terminally modified MHC class I peptides (i.e. 4-pentynoyl-FFRK-NLVPMVATV for α-GalCer-pp65_495-503_, 4-pentynoyl-FFRK-RAHYNIVTF for α-GalCer-E7_49-57_ and 4-pentynoyl-PLTK-GILGFVFTL for α-GalCer-M1_58-66_)^25^ utilizing copper catalyzed alkyne-azide cycloaddition methodology^50^. The vaccines were purified by the use of preparative HPLC (Phenomenex Luna C18(2), 5 µm, 250 × 30 mm, 30 °C, 40 mL/min) with a TFA modified water-MeOH mobile phase. Analytical data: α-GalCer-E7_49-57_, HRMS-ESI m/z calculated for C_162_H_260_N_32_O_33_ [M + 2H]^2+^ 1590.9826, found 1590.9823; α-GalCer-M1_58-66_, HRMS-ESI m/z calculated for C_150_H_252_N_23_O_32_ [M + H]^+^ 2887.8799, found 2887.8804. The peptides 4-pentynoyl-FFRK-RAHYNIVTF (E7_49-57_), 4-pentynoyl-PLTKGILGFVFTL (M1_58-66_) and 4-pentynoyl-FFRK-NLVPMVATV (pp65_495-503_) were purchased from Peptides & Elephants GmbH (Potsdam, Germany).

### Solubilization and administration of compounds for biological studies

Solubilization of α-GalCer and vaccines was achieved by freeze-drying the samples in the presence of sucrose, L-histidine, and Tween 20 as previously described^51^. All solubilized compounds were diluted in sterile water before use *in vitro*.

### Isolation of human PBMCs

Venous blood was drawn into heparin-containing tubes, diluted 1:1 in phosphate buffered saline (PBS; Gibco), and PBMCs isolated by density centrifugation (Lymphoprep™; Axis-Shield, Oslo, Norway). PBMCs were washed twice and either used fresh or cryopreserved in 10% dimethyl sulfoxide (Sigma-Aldrich) and 90% fetal bovine serum (FBS; Gibco) and thawed immediately before use. All donors gave written informed consent; this study was approved by the Human Ethics Committee of Victoria University, and all experiments were performed in accordance with relevant guidelines and regulations.

### Cell-free CD1d presentation assay

Dependence of NKT cell activation on proteolytic cleavage of α-GalCer-pp65_495-503_ was tested in a cell-free assay. Flat-bottomed 96-well tissue culture plates were coated with 5 μg/mL mouse CD1d monomers (NIH Tetramer Core Facility) overnight at 4 °C. Equimolar concentrations of the vaccine α-GalCer-pp65_495-503_ and free α-GalCer were pre-treated with cathepsin-B (Sigma-Aldrich) or mock-treated with PBS for 24 h, at 37 °C, before the equivalent of 50 ng/digest was added to the coated plate for 3 h, at 37 °C. Plates were then washed with Iscove’s Minimum Essential Medium (IMDM; Gibco). DN32.D3 NKT hybridoma cells^52^ were seeded at a density of 3 × 10^4^ cells/well and the plate was incubated at 37 °C in IMDM supplemented with 5% fetal bovine serum (FBS) (Sigma-Aldrich), 2 mM glutamax, 100 U/mL penicillin, 100 mg/mL streptomycin and 50 mM 2-mercaptoethanol (all Invitrogen). The following day, supernatants were collected and IL-2 concentration determined by ELISA (BioLegend), according to the manufacturer’s instructions.

### Generation of monocyte-derived (mo)DC

Immature DC were generated from human PBMCs over 6 days from adherent monocytes using RPMI (Gibco) supplemented with 2% autologous human serum containing 1000 U/mL GM-CSF and 1000 U/mL IL-4. Cytokines were re-added after 3 days in culture. For co-culture experiments to assess DC activation by flow cytometry, 5 × 10^4^ DCs/well were cultured in low adherence 96-well plates (Nunc) with 5 × 10^3^ flow-sorted human NKT cells/well in the presence of 1 µM of α-GalCer-pp65_495-503_ or α-GalCer.

### Flow cytometric analysis

The following antibodies (Ab) were used: anti-CD209 (DCN46; BD), anti-CD83 (HB1SE; Biolegend), anti-CD86 (IT2.2; BioLegend), anti-CD3 (UCHT1; BioLegend), anti-CD19 (HIB19; BioLegend), anti-CD8 (RPA-T8; BioLegend), anti-Vα24 Jα18 TCR (6B11; Biolegend), anti-CD137 (4B4-1; BioLegend), anti-Ki67 (Ki67; BD Pharmingen), PE-HLA-A*0201 NLVPMVATV and APC-HLA-A*0201 GILGFVFTL (both Immudex). Cells were stained for 15 min at room temperature, washed 2 × in flow buffer (PBS + 5% FBS) and fixed in 1:1 4% formalin:PBS before analysis. In some experiments (Fig. 3A–F), MHC class I dextramers PE-HLA-A*0201 NLVPMVATV or PE-HLA-A*0201 GILGFVFTL (Immudex) were added after live/dead staining, for 10 min prior to surface Ab staining. All flow cytometry was performed on a BD Biosciences LSRFortessa or LSRII SORP flow cytometer. Cells were stained with Fixable Live/Dead NIR (Invitrogen). Data analysis was performed using FlowJo software (Tree Star, Inc.). For all analyses, dead cells and doublets were first excluded by gating.

### Intracellular cytokine staining

For intracellular cytokine staining of peptide-specific CD8^+^ T cells (Fig. 4E), anti-CD29/49d (BD Biosciences) and anti-CD107a (H4A3; BioLegend) were added to the cells 1 h before addition of 300 ng/mL monensin and 500 ng/mL brefeldin-A (both Sigma-Aldrich). After 18 h in culture, live/dead and surface Ab staining was performed before cells were fixed in Cytofix/Cytoperm™ solution (BD Biosciences) for 20 min. Intracellular cytokine staining was then performed using anti-IFN-γ (clone 45.B3; BioLegend) for 30 min at room temperature. Cells were washed 2× in PermWash™ buffer (BD Biosciences) and PBS before analysis. For intranuclear staining of NKT cells with anti-Ki67 (Fig. 1D), following live/dead and surface Ab staining, cells were permeabilized and fixed in FoxP3 Perm/Fix buffer (BioLegend) at room temp for 20 min. Cells were washed and re-suspended in FoxP3 Perm buffer (BioLegend) for anti-Ki67 staining for 30 min at room temperature, then washed 2× in flow buffer before analysis.

### NKT cell expansion *ex vivo*

For co-culture experiments, NKT cells were sorted from PBMCs isolated from a healthy human donor with a resting NKT cell frequency of 1% of CD3^+^ T cells. Briefly, 10 × 10^6^ PBMCs/mL were incubated with anti-CD3, anti-CD19 and anti-Vα24 Jα18 TCR (6B11) for 30 min on ice. Cells were washed 2× in sterile flow buffer and re-suspended in 2 mL sterile flow buffer containing 2.5 µg/mL DAPI. The DAPI^low^ CD3^+^ CD19^−^ 6B11^+^ cells were then collected using a BD Biosciences Influx Cell Sorter. Cells were seeded into a 96-well plate at 5 × 10^3^ cells/well in IMDM supplemented with 5% human AB serum (Sigma-Aldrich), 2 mM glutamax, 100 U/ml penicillin and 100 mg/mL streptomycin and 50 mM 2-mercaptoethanol (complete IMDM; cIMDM), and containing 1 μg/mL PHA (Sigma-Aldrich) plus 100 U/mL IL-2 (Peprotech) and 10^5^ irradiated (50 Gy) PBMC feeder cells. Fresh cIMDM containing 100 U/mL IL-2 was added every 3-4 days. Feeder cells were replaced every 10 days. Before use, dead cells were removed using the MACS Dead Cell Removal Kit (Miltenyi Biotec).

### Analysis of *in vitro* human NKT cell activation

PBMCs from a HLA-A*02-negative healthy human donor were cultured in cIMDM at 3 × 10^5^ cells/well with 1 μM α-GalCer or molar equivalent of α-GalCer-pp65_495-503_ in the presence of 50 μg/mL LEAF-pure anti-CD1d (clone 51.1; BioLegend) or matched isotype control. For 7 day cultures (Fig. 1C), 50 U/mL of IL-2 was added on day 2. IFN-γ secretion was assessed by Elispot as described below.

### ELISpot assay

IFN-γ production was quantified after 24 h using a human IFN-γ ELISpot kit (Mabtech), according to manufacturer’s instructions. Briefly, Millipore MultiScreen-HA 96-well filter plates (Millipore) were coated with 5 μg/mL IFN-γ mAb in PBS overnight at 4 °C. Cells were seeded at 2 × 10^5^ cells/well in cIMDM and incubated with the relevant compounds overnight. After washing with PBS, plates were incubated with biotin-labeled anti-IFN-γ for 1 h at room temperature. Plates were then washed and treated with 1:1000 dilution of streptavidin-alkaline phosphatase (Sigma) for 30 min at room temperature. Plates were developed with nitro-blue tetrazolium and 5-bromo-4-chloro-3-indolyl-phosphate substrate (Mabtech) until all spots were clearly visible. Developed plates were dried and counted on an automated ELISpot reader (Autoimmun Diagnostika, Strassberg, Germany). Cells treated with PHA were used as a positive control.

### Stimulation of human CMV-specific CD8^+^ T cells *in vitro*

PBMCs from HLA-A*02 positive CMV-seropositive donors were cultured cIMDM at 3 × 10^5^ cells/well for 5–7 days with pp65_495-503_ peptide, admixed peptide and α-GalCer, or the α-GalCer-pp65_495-503_ vaccine all at equimolar concentrations of 1 µM.

### Depletion of NKT cells

PBMCs from HLA-A*02 positive CMV-seropositive donors were incubated at 4 °C for 10 min with anti-Vα24 Jα18 TCR biotinylated antibody (clone 6B11; eBioScience). Cells were then washed twice in Würzburger buffer and incubated at 4 °C, gently shaking, with twice the recommended concentration of biotin binding beads (Dynabead; Invitrogen) for 20 min. Unbound cells were isolated using a DynaMag-15 magnet (ThermoFisher). This process was repeated three times to ensure >99% of NKT cells were depleted from the PBMCs.

### RNA isolation and Nanostring analysis

After seven days in culture as detailed above (“Stimulation of human CMV-specific CD8^+^ T cells *in vitro*”), RNA was extracted from PBMCs derived from four donors using the RNeasy Micro Kit (Qiagen) according to the manufacturer’s instructions. RNA purity and integrity were assessed using a Nanodrop spectrophotometer (ThermoFisher) and gel electrophoresis. Nanostring RNA analysis of 700 immune-related genes was performed using the nCounter GX Human PanCancer Immune profiling Kit (XT) on the nCounter® Analysis System. Data were analyzed using the nSolver Analysis software package and Microsoft Excel. Default normalization settings were used for all data. The online tool CIMminer (https://discover.nci.nih.gov/cimminer/home.do) was used to generate heat maps.

### *In vivo* cytotoxicity assay

Conjugate vaccines were diluted in sterile PBS for injection. Animals were injected intravenously with 8.5 μg of α-GalCer-E7_49-57_ conjugate vaccine, equimolar concentrations of α-GalCer and E7_49-57_, an equimolar concentration of E7_49-57_ peptide alone, or PBS control. After 10 days, syngeneic splenocytes were loaded with 10 μM, 1 μM or 0.1 μM E7_49-57_ peptide and labeled with 2.5 μM, 0.5 μM, or 0.1 μM carboxyfluorescein succinimidyl ester (Life Technologies), respectively. A control target population without antigen was labeled with 20 μM chloro-methyl-benzoyl-aminotetramethyl-rhodamine (Life Technologies). A mixture of the four target cell populations was injected intravenously into immunized mice, and specific lysis of peptide-loaded targets was assessed 18 h later by flow cytometric analysis of peripheral blood. Survival of peptide-pulsed targets was calculated relative to that of the control target, and cytotoxic activity was expressed as percent specific lysis.

### Analysis of anti-tumor activity *in vivo*

The mouse lung cancer cell line TC-1, a mouse lung epithelial cell line transformed with HPV16-E6 and -E7 genes^53^, was cultured in RPMI1640 supplemented with 10% FBS, 100 U/ml penicillin, 100 mg/ml streptomycin (all from Gibco), and 500 mg/ml G418 (Sigma), and resuspended in PBS for subcutaneous injection. Groups of naive C57BL/6 mice (n = 5) were implanted with 2.5 × 10^5^ cells in the flank. On day 8, when tumors reached at least 5mm in diameter, mice were given one intravenous treatment with either 1.5 µg (0.47 nmol) of α-GalCer-E7_49-57_ conjugate vaccine, an admix of 100 ng (0.09 nmol) HPV16 E7_49-57_ peptide and 5 nmol of α-GalCer, HPV16 E7_49-57_ peptide alone or α-GalCer alone; the concentrations of α-GalCer and peptide used were selected as the optimal concentrations based on *in vivo* titrations. Some mice received two doses of the α-GalCer-E7_49-57_ with a week interval. A control group was injected with PBS. Tumor growth was monitored three times per week, with tumor size calculated as the product of the two bisecting diameters. Measurements were stopped for each group when the first mouse developed a tumor exceeding 400 mm^2^. The C57BL/6 mice used were purchased from Animal Production Colonies, Frederick Cancer Research Facility, National Cancer Institute. All animal experiments were approved by the Animal Care and Use Committee of the National Cancer Institute, and all experiments were performed in accordance with relevant guidelines and regulations.

### Statistical analysis and data availability

All statistical analysis was performed using GraphPad Prism software (GraphPad Prism Inc). For Fig. 1B,D and 2F, two-way analysis of variance (ANOVA) was performed before the Bonferroni multiple comparisons test. For Fig. 1E, the Mann-Whitney U test was used. For Fig. 2B, D, the Friedman test was used, with Dunn’s multiple comparisons. In all experiments, *P* values ≤ 0.05 were taken to be significant. The data generated during the current study are available from the corresponding author on reasonable request.

## Electronic supplementary material

Supplementary data
