# Supplementary material for: Glycolipid-peptide conjugate vaccines enhance CD8+ T cell responses against human viral proteins
Source: Sci Rep. 2017 Oct 27;7:14273. doi: 10.1038/s41598-017-14690-5 (PMC5660197; doi:10.1038/s41598-017-14690-5)
Supplement: Supplementary file 1 — Supplementary data [file 41598_2017_14690_MOESM1_ESM.pdf]

**Supplementary Data; Supplementary Methods**

**Glycolipid-peptide conjugate vaccines enhance CD8$^+$ T cell responses against human viral proteins**

Speir M, Authier-Hall A, Brooks CR, Farrand KJ, Compton BJ, Anderson, RJ, Heiser A, Osmond TL, Tang CW, Berzofsky JA, Terabe M, Painter GF, Hermans IF, Weinkove R

**Supplementary Figure S1:** C57BL/6 mice (n = 5 per group) were vaccinated intravenously with α-GalCer + $E7_{49\text{-}57}$ peptide admix, α-GalCer-$E7_{49\text{-}57}$ or PBS as a naïve control. Splenocytes from vaccinated mice were re-stimulated ex vivo with $E7_{49\text{-}57}$ peptide and production of IFN-γ was assessed by ELISpot; *** p<0.001, * p<0.05; SFU, spot-forming units.

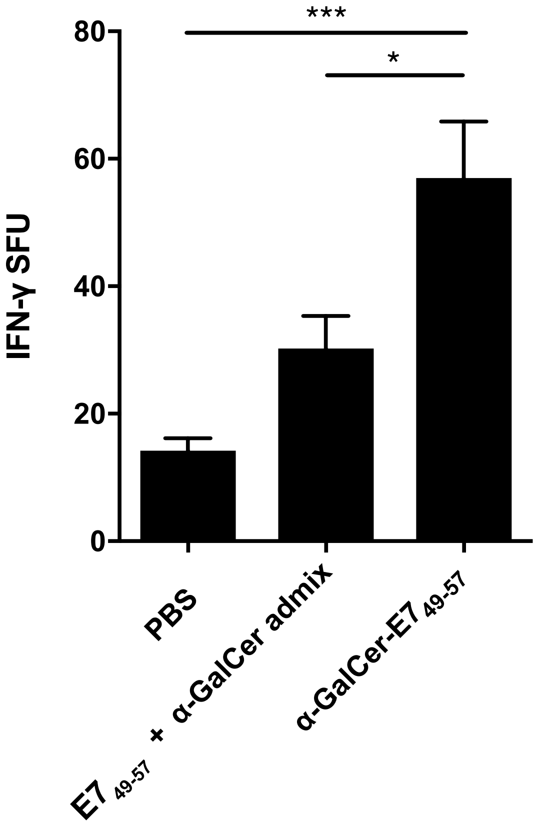

**Supplementary table S1:** Expression of the 30 immune-related genes most upregulated upon culture of human PBMCs with α-GalCer-pp65$_{495\text{-}503}$, compared to pp65$_{495\text{-}503}$ peptide alone. Mean of four human donors.

| Gene | Mean expression (relative to peptide alone) |
|---|---|
| *CXCL10* | 16.88 |
| *CCL13* | 5.86 |
| *SPINK5* | 5.44 |
| *CXCL11* | 5.04 |
| *C8B* | 4.66 |
| *CCL1* | 3.31 |
| *CXCL9* | 3.20 |
| *CCL8* | 2.96 |
| *TNFRSF8* | 2.85 |
| *IFNG* | 2.71 |
| *COLEC12* | 2.66 |
| *TFEB* | 2.49 |
| *IDO1* | 2.29 |
| *IL3RA* | 2.24 |
| *IFIT2* | 2.24 |
| *TREM1* | 2.21 |
| *DUSP4* | 2.18 |
| *C9* | 2.14 |
| *FEZ1* | 2.09 |
| *MRC1* | 2.06 |
| *IL2RA* | 2.06 |
| *CTLA4* | 2.05 |
| *LCN2* | 1.96 |
| *RORC* | 1.95 |
| *IL1R1* | 1.93 |
| *TNFRSF9* | 1.90 |
| *GZMB* | 1.89 |
| *CCL24* | 1.82 |
| *MEFV* | 1.79 |
| *SERPINB2* | 1.78 |

## Supplementary Methods

### *Ex vivo* peptide-specific IFN-$\gamma$ production

Conjugate vaccines or admixed controls were diluted in sterile phosphate buffered saline (PBS) solution for injection. C57BL/6 mice were injected intravenously with either 8.5 $\mu$g of $\alpha$-GalCer-E7$_{49-57}$ conjugate vaccine, admixed 2.3 $\mu$g $\alpha$-GalCer and 3 $\mu$g HPV16 E7$_{49-57}$ peptide (each corresponding to 2.68 nmol), or PBS alone as a naïve control. After 11 days, spleens were harvested for quantitation of *ex vivo* IFN-$\gamma$ production using a mouse IFN-$\gamma$ ELISpot$^{PLUS}$ kit (Mabtech), according to manufacturer's instructions. Briefly, Millipore MultiScreen-HA 96-well filter plates (Millipore) were coated with 15 $\mu$g/mL IFN-$\gamma$ mAb in PBS overnight at 4 °C. Splenocytes were seeded at 4 x $10^5$ cells/well and incubated overnight with 0.1$\mu$M E7$_{49-57}$ peptide at 37°C. Following washing, plates were incubated with 1 $\mu$g/ml biotin-labeled anti-IFN-$\gamma$ for 2 h at room temperature. Plates were then washed and treated with 1:1000 dilution of streptavidin-alkaline phosphatase for 1 h at room temperature. Plates were developed with nitro-blue tetrazolium and 5-bromo-4-chloro-3-indolyl-phosphate substrate until all spots were clearly visible. Developed plates were dried and counted on an automated ELISpot reader (Autoimmun Diagnostika, Strassberg, Germany).
